# Assessment of Gallbladder Drainage Methods in the Treatment of Acute Cholecystitis: A Literature Review

**DOI:** 10.3390/medicina60010005

**Published:** 2023-12-20

**Authors:** Dorotea Bozic, Zarko Ardalic, Antonio Mestrovic, Josipa Bilandzic Ivisic, Damir Alicic, Ivan Zaja, Tomislav Ivanovic, Ivona Bozic, Zeljko Puljiz, Andre Bratanic

**Affiliations:** 1 Department of Gastroenterology, University Hospital of Split, Spinciceva 1, 21000 Split, Croatia; zardalic@gmail.com (Z.A.); antonio.mestrovic1@gmail.com (A.M.); dalicic@gmail.com (D.A.); ivan.zaja@yahoo.com (I.Z.); zpuljiz4@gmail.com (Z.P.); andrebratanic@net.hr (A.B.); 2 Department of Gastroenterology, General Hospital of Sibenik-Knin County, Stjepana Radica 83, 22000 Sibenik, Croatia; josipa.bilanzic0000@gmail.com; 3 University Department of Health Studies, University of Split, Rudjera Boskovica 35, 21000 Split, Croatia; 4 Department of Abdominal Surgery, University Hospital of Split, Spinciceva 1, 21000 Split, Croatia; tomo.mefst@gmail.com; 5 Department of Rheumatology and Immunology, University Hospital of Split, Spinciceva 1, 21000 Split, Croatia; ivona.bozic.7@gmail.com; 6 School of Medicine, University of Split, Soltanska 2, 21000 Split, Croatia

**Keywords:** acute cholecystitis, percutaneous gallbladder drainage, percutaneous cholecystostomy, endoscopic gallbladder drainage, transpapillary gallbladder drainage

## Abstract

Gallbladder drainage is a treatment option in high-risk surgical patients with moderate or severe acute cholecystitis. It may be applied as a bridge to cholecystectomy or a definitive treatment option. Apart from the simple and widely accessible percutaneous cholecystostomy, new attractive techniques have emerged in the previous decade, including endoscopic transpapillary gallbladder drainage and endoscopic ultrasound-guided gallbladder drainage. The aim of this paper is to present currently available drainage techniques in the treatment of AC; evaluate their technical and clinical effectiveness, advantages, possible adverse events, and patient outcomes; and illuminate the decision-making path when choosing among various treatment modalities for each patient, depending on their clinical characteristics and the accessibility of methods.

## 1. Introduction

Acute cholecystitis (AC) is an acute inflammation of the gallbladder that develops promptly within a few hours and is usually the result of cystic duct obstruction with the gallstone or sludge. The incidence is estimated at 6300 per 100,000 individuals under the age of 50 and 20,900 per 100,000 individuals over the age of 50 [1]. Approximately 20% of patients with gallstones will develop gallstone-related complications, with an annual incidence of 1–4%. Acalculous cholecystitis accounts for 5–10% of patients with AC, develops on the basis of bile stasis or gallbladder wall ischemia, and is usually associated with critical illness, diabetes, human immunodeficiency virus (HIV), vasculitis, or total parenteral nutrition [2]. 

The clinical presentation of AC may be mild, self-limited illness, but also fulminant and potentially life-threatening disease. According to the Society of Interventional Radiology, successful treatment of AC is defined by the resolution of clinical symptoms, including fever and pain, and a decrease in inflammatory parameters [3]. Initial management includes the assessment of vital signs, intravenous fluid replacement, establishment of electrolyte balance, and administration of analgesics and antibiotics [4]. Tokyo Guidelines 2018 (TG18) recommend various treatment approaches depending on the severity of AC [5]. Patients with grade I (mild) AC should be treated with laparoscopic cholecystectomy (Lap-C) at an early stage, within 3–7 days of the symptom onset. In patients with grade II (moderate) AC, Lap-C is also the treatment of choice if the patient’s performance status allows it and if the advanced Lap-C technique is available. If the patient’s condition is poor, early biliary drainage and/or delayed Lap-C may be selected. Patients with grade III AC and/or high surgical risk should be treated with early drainage. In patients with good performance status, no signs of organ failure, or negative predictive factors, early Lap-C at the advanced center may also be chosen as the treatment modality [6].

TG18 stated that percutaneous transhepatic gallbladder drainage (PT-GBD) should be recommended as the first alternative to cholecystectomy in patients with high surgical risk. Another alternative procedure is endoscopic gallbladder drainage, which includes endoscopic transpapillary gallbladder drainage (ET-GBD) via endoscopic retrograde cholangiopancreatography (ERCP) and the endoscopic ultrasound-guided gallbladder drainage (EUS-GBD) [7]. World Society of Emergency Surgery (WSES) guidelines recommend gallbladder drainage for septic patients who are not suitable for surgery. Additionally, they consider endoscopic methods of biliary drainage safe and effective alternatives to percutaneous methods if they are performed in high-volume centers by skilled endoscopists [8].

**Scheme 1:**
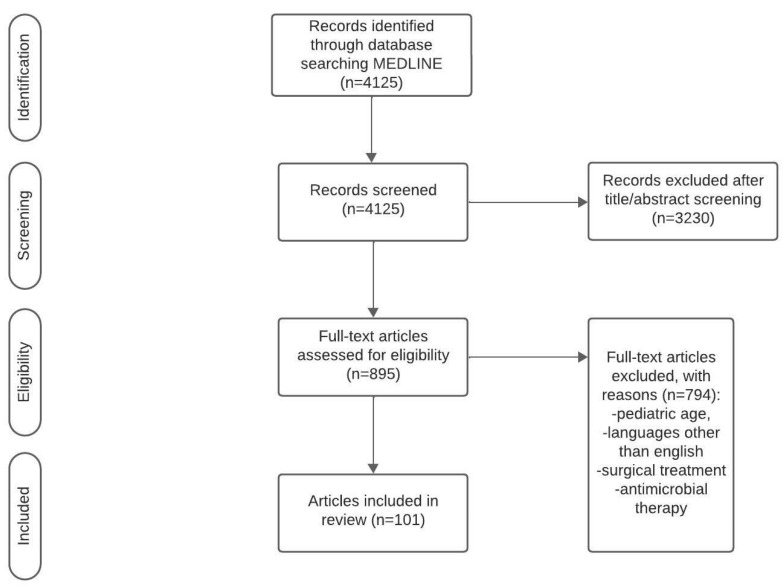
Flow diagram.

The aim of this paper is to present currently available percutaneous and endoscopic gallbladder drainage methods in the treatment of AC and to evaluate and compare their effectiveness, possible adverse events, and patient outcomes. The MEDLINE database was systematically searched for the relevant literature, concluding on 25 October 2023. A total of 101 publications relevant to the topic were identified (Scheme 1). 

## 2. Percutaneous Gallbladder Drainage

Percutaneous gallbladder drainage (PGBD) or percutaneous cholecystostomy (PCT) is, by definition, a therapeutic procedure that implies sterile placement of a needle into the gallbladder using the imaging guidance, followed by the aspiration of bile, and is eventually completed with the placement of a tube for external drainage of the gallbladder contents [3]. Among several methods available for the purposes of gallbladder drainage, multislice computed tomography (MSCT)-guided PT-GBD is by far the most commonly implemented in clinical practice. Originally described in 1979. by Elyaderany et al. as the treatment modality for obstructive jaundice, it was first introduced for the management of AC by Radder in 1982 [9,10]. The other, more available and practical PGBD modality that enables real-time imaging is ultrasound (US)-guided gallbladder drainage. It enables bedside performance and excludes radiation exposure. 

### 2.1. Indications and Contraindications

**Figure 1:**
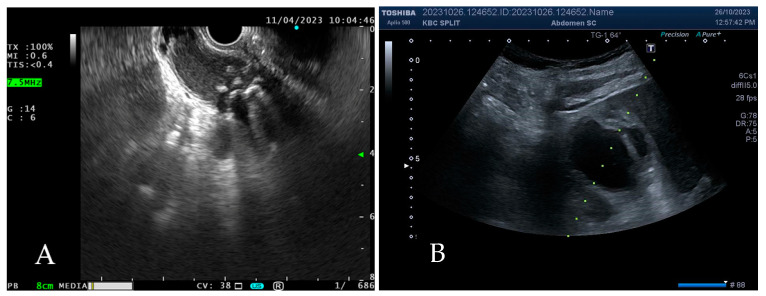
Calculous and acalculous cholecystitis. EUS-obtained image of the acute calculous cholecystitis (**A**) and the US image of the acalculous cholecystitis with the proposed US-guided transhepatic needle pathway (**B**).

PGBD is indicated in patients treated for AC with high anesthetic risk (American Society of Anaesthesiologists grade III (ASA III)) due to comorbidities or advanced age but also in the failure of antibiotic treatment, severe sepsis, suspected gallbladder perforation or empyema, and lastly, when the patient refuses surgical treatment [11]. Since gallbladder distension and inflammation are usually caused by cystic duct obstruction, PGBD acts via gallbladder decompression and consequently promotes the migration of gallstones that block the entrance into the cystic duct (Figure 1A). Apart from the management of calculous cholecystitis, PGBD is also used as the treatment modality for acalculous cholecystitis, with reported indication rates from 1.6 to 52% (Figure 1B) [11]. 

Contraindications for PGBD include the presence of ascites, coagulopathy, an allergy to iodinated contrast, an interposed bowel, an inaccessible gallbladder, or a gallbladder filled with calculi [12,13]. Regarding ascites, paracentesis may be performed prior to or during the PGBD, although a recent retrospective review that included 255 patients (97 with ascites and 158 without) found no difference (*p* = 0.834) in complication rates between the groups [14]. 

### 2.2. Procedure

Preprocedural assessment includes the coagulation profile tests (prothrombin time (PT), international normalized ratio (INR), and platelets) and correction with the blood products if necessary. The timely withdrawal of anticoagulants and/or antiaggregants is mandatory, followed by the low molecular weight heparin (LMWH) bridging in patients with high thrombotic risk or application of antidots in an urgent setting. Patients are generally treated with intravenous antibiotics depending on the local resistance.

MSCT-guided PGBD is performed by an interventional radiologist who decides on the approach and route according to their personal preferences and patient characteristics. US-guided PGBD may be performed by a radiologist or gastroenterologist using the convex probe at a frequency range of 2.5–6 MHz, while a sector probe (2–5 MHz) is preferable for the intercostal approach. The patient is placed in a supine position, and following the skin asepsis and local anesthesia (by subcutaneous administration of lidocaine), the puncture is performed [15]. Preprocedural premedication (fentanyl, midazolam) is optional. The gallbladder may be reached subcostally or intercostally. A subcostal approach is preferred whenever possible, but if the intercostal direction is required (due to the gallbladder position), special attention must be paid to avoid puncturing the pleural space (diaphragm serves as orientation), aiming to pass superior to the rib, thus avoiding the intercostal neurovascular bundle [16]. 

**Figure 2:**
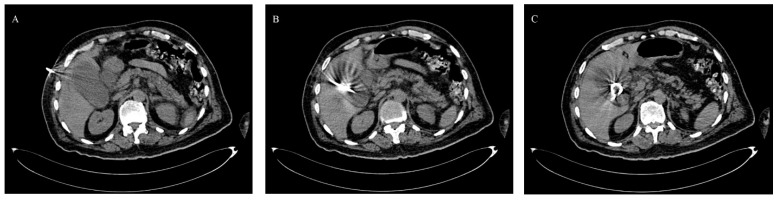
MSCT-guided gallbladder drainage. MSCT-guided gallbladder drainage using the transhepatic approach: (**A**) needle introduction, (**B**) insertion into the gallbladder, and (**C**) guidewire with J tip positioned into the gallbladder.

The placement of catheters includes transhepatic and transperitoneal routes [3,9,17,18]. The transhepatic route is used more commonly since it provides anatomical fixation and greater stability of the catheter due to direct penetration of the liver (Figure 2). Additionally, liver parenchyma tamponades the gallbladder wall and thereby reduces bile leakage [17,18,19]. Contrary, the transperitoneal route bypasses the liver and, therefore, has priority in patients with coagulopathy or diffuse liver disease. It is also a reasonable choice when the distended gallbladder directly adheres to the abdominal wall [20]. Clinical studies showed contradictory results regarding the safety of these routes. While certain authors find the transperitoneal route insecure due to the reported higher risk of bile leakage, other authors found no significant differences between the methods [20,21,22]. A recent meta-analysis that included 684 patients compared the aforementioned routes and found a significantly higher risk of bleeding (6.3% vs. 1.6%, OR 4.02, *p* = 0.004) when using the transhepatic route [23]. The decision should be left to the discretion and experience of the radiologist. 

**Figure 3:**
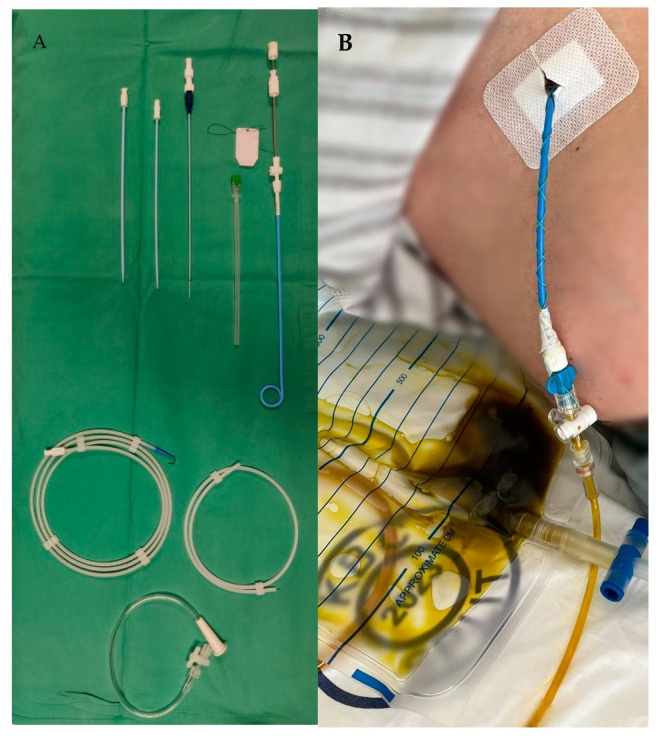
Percutaneous gallbladder drainage set and postprocedural view. (**A**) Pigtail locking set includes (from left to right and from top to bottom) three dilators of different widths, introducer needle with stylet, metal stiffening cannula with the biliary drainage catheter, stainless steel J-tip guidewire, stainless steel guidewire with floppy tip, and device to connect catheter to the drainage bag; (**B**) postprocedural presentation of the drainage catheter and the drainage bag.

The catheter placement is performed using one of the two available techniques: the modified Seldinger (multiple-step) technique or the trocar (one-step) technique [15]. Seldinger technique is widely used in conjunction with the transhepatic approach: following the gallbladder puncture with an access needle (22–18 gauge), a guidewire is inserted to allow for the gradual introduction of dilatators, which then enables further insertion of the locking pig-tail catheter (Figure 3A) [13]. After the tip of the catheter enters the gallbladder, the catheter is unlocked from the inner stiffener and advanced over the wire into the gallbladder [15]. The wire is then removed, and the catheter is locked in position for gravity drainage (Figure 3B). The usual catheter diameter is 7–10 French and contains multiple holes that provide adequate bile drainage [12]. After placing the drain, bile is sent for microbiological analysis. Medical staff, as well as patients, are recommended to flush the catheter with 5–15 mL of sterile saline every 12 h to prevent the occlusion with viscous bile, mud, or blood clots [24]. Postprocedurally, patients are monitored for at least six hours. The other technique, known as the ‘trocar’ technique, enables the direct insertion of the catheter and usually accompanies the transperitoneal approach [12,13].

By using a fine needle, the Seldinger technique reduces the potential risk of perforation of a neighboring organ and, thus, is considered a safer and more favorable option than the trocar technique [15,16,25]. However, although results of a prospective study that compared the US-guided trocar technique and the fluoroscopy-guided Seldinger technique showed their equal effectiveness, the US-guided trocar technique was associated with fewer procedure-related complications, required less procedural time, and resulted in decreased postprocedural pain [26]. 

Although the pigtail catheter is the standard of use, some authors propose using the central venous catheter (CVC) [27]. However, CVC is shorter (20 cm vs. 39 cm) and has a higher risk of migration due to the lack of a locking system and internal fixation. Moreover, its small internal diameter often obstructs the adequate drainage of thick bile [15].

### 2.3. Timing and Duration

When discussing the PGBD timing, authors agree that the best timing is the earliest one. Namely, early PGBD conducted less than 24 h after the symptom onset reduces hospital stay and procedure-related complications compared to the postponed approach [12,28]. Additionally, Bickel et al. found that the PGBD conducted within the first two days from the symptom onset is associated with a lower incidence of conversion from laparoscopic to open cholecystectomy, presumably due to the prevention of fibrosis and adhesion formation [11,29]. 

Although certain authors support the late drain removal, this doctrine is, as of late, being replaced with the early drain removal immediately after the AC resolution since significant clinical and laboratory improvement has been detected in 86.7% of patients four days after the procedure [17,30,31]. Macchini et al. conducted a systematic review and found that the PGBD duration does not affect a short-term clinical outcome [32]. Hung YL et al. stated three main reasons supporting the early drain removal: mature tract that prevents bile leakage reported good clinical outcomes, and prolonged drainage is considered a precipitating factor for recurrent biliary events. They recommend performing the clamping test 24–48 h before the catheter removal [17]. Bundy J et al. conducted a retrospective study that included 324 patients treated with PGBD from 2010 to 2017 with a mean tube indwelling time of 89 (range 0–586) days. Only 1% of patients had mild complications, and the reported long-term mortality rate was 6.8% [33]. Contrary, Simunic et al. performed a retrospective study that included 92 patients treated with PGBD from 2015 until 2020 with a significantly shorter average drainage duration of 10.1 ± 4.8 (3–28) days, complication rate of 6.5%, and intrahospital mortality of 10% [34]. In order to compare these strategies, Di Martino et al. conducted a retrospective comparative study on 151 patients who were separated into two groups depending on the PGBD duration (median 52 vs. 8 days) with no difference in complication rates, but a significantly higher risk of recurrent disease and readmissions in the group with the early drain removal [35]. On the other side, Wang CH et al. evaluated the long-term outcomes of 184 patients treated with PTGBD with a one-year follow-up. The authors reported a one-year recurrence rate of 9.2% and found a positive correlation of AC recurrence with the PGBD duration >32 days [36]. Other authors reported even higher rates of AC recurrence (25–40%), which usually occur within the first year after the procedure [37]. To avoid recurrent AC following the PGBD, Hasbahceci M. et al. recommend keeping the catheter in place until the time of surgery in surgically fit patients [38]. In certain clinical scenarios that slow tract maturation, such as unregulated diabetes, long-term steroid therapy, malnutrition, or ascites, it is also recommended to leave the drain in place for a longer period [16,25].

Prolonged PGBD results in longer hospitalization, is related to a higher risk of PGBD dysfunction, and therefore, inevitably leads to higher expenses, not to neglect the patient discomfort [34]. We propose the early drain removal as soon as the clinical status and laboratory tests indicate the inflammation withdrawal but with the recommendation of the earliest possible cholecystectomy in all the surgically fit patients due to a high risk of recurrent disease or other potentially detrimental biliary complications such as acute pancreatitis, cholangitis, or biliary sepsis. 

### 2.4. Adverse Events and Outcomes

The reported incidence of PGBD-related adverse events varies significantly between the studies and is reported in a wide range (2.5–69%). However, the most commonly reported are mild complications with a rate of 5–15% [12,17,34,39,40,41] and include catheter dislodgement or obstruction, minor bleeding, bile leakage, and vasovagal reaction [18].

Severe complications such as major bleeding, bowel perforation, pneumothorax, or death are rarely reported [17,20,24,30,34,36,42,43]. Catheter dislodgement is usually caused by respiratory movement and feeding-related gallbladder contractions, while bleeding might be caused by a fragile and inflamed gallbladder wall or intrahepatic vascular trauma [37]. The slight pericatheter bile leak may be present immediately post-procedurally and usually resolves spontaneously, while the significant leak may cause biliary peritonitis and require prompt surgical treatment [37]. A cholecystocutaneous or cholecystohepatic fistula is a rare complication that may cause persistent leakage after the drain removal. Control cholangiography with optional revision is performed in cases of biliary leakage, catheter dislodgement, or catheter obstruction. Minor bleeding is usually treated conservatively, while major bleeding may be treated either by interventional radiologists using coiling or embolization techniques or surgically [17]. 

Published studies have reported a short-term mortality rate of 5.4–35.8%, which is generally related to patient comorbidities and not the procedure itself [24,28,30,31,32,33,34,44,45,46]. Predictions of worse outcomes include high Acute Physiology and Chronic Health Evaluation score II (APACHE II) and Charlson comorbidity index (CCI), as well as late procedure timing, as shown in one retrospective analysis [40]. Accordingly, a systematic review that included 46 studies and 312,085 patients proved PGBD to be a successful and safe treatment modality for AC and found only four PGBD-related fatal outcomes (mortality 0.001%) [11]. 

Regarding the postprocedural cholecystectomy, certain authors recommend surgery in the same hospital admission; some consider the ideal time for surgery 7–26 days following the procedure, while certain surgeons are prone to wait at least 3 months for the complete regression of the gallbladder inflammation [37]. Whether to perform the postprocedural cholecystectomy mainly depends on the patient’s clinical condition after the acute inflammation withdrawal. If the patient is fit for surgery, the majority of authors support interval cholecystectomy due to the high risk of recurrent biliary events usually caused by cystic duct obstruction or bile stasis and, consequently, high medical expenses [17,47,48]. 

## 3. Endoscopic Gallbladder Drainage

Besides the extensive experience and a high rate of clinical success of PGBD, its contraindications and complications limit its use, especially in the long term [49,50].

Therefore, endoscopic techniques of gallbladder drainage emerge as the more appropriate choice in a significant proportion of patients unfit for surgical treatment. These include ET-GBD and EUS-GBD [49,51].

### 3.1. Endoscopic Ultrasound-Guided Gallbladder Drainage 

EUS-GBD comprises a stent placement into the gallbladder from the stomach or duodenum under the EUS guidance. The implementation of EUS in this indication was first reported in 2007 [52].

#### 3.1.1. Indications and Contraindications

EUS-GBD is currently used as a replacement for PGBD in high-risk surgical patients when prolonged drainage is required [53]. Contraindications include local findings that interfere with the EUS-GBD performance or greatly increase its risk (e.g., gallbladder perforation, biliary peritonitis, or the presence of large-volume ascites). In addition, there are some contraindications for the procedure on a general level, such as coagulopathy, thrombocytopenia, or inability to undergo anesthesia [54,55].

#### 3.1.2. Procedure

Initially, double pigtail plastic stents and biliary self-expandable tubular stents were used. However, due to the risk of bile leakage caused by their length and the suboptimal adherence to the luminal walls and the lack of flanges that resulted in frequent stent migration, they proved not to be an optimal solution. Further improvement occurred with the development of lumen-apposing metal stents (LAMS). Its use in gallbladder drainage was first reported in 2015 [56]. LAMS offered a better solution due to a tight apposing of both the gallbladder and the gastrointestinal luminal walls, flanges that prevent migration, and wider inner diameters, which allow for the evacuation of gallstones or even therapeutic cholecystoscopy [57,58,59,60,61]. As a result of these advantages, they considerably replaced plastic and endobilliary self-expanding stents.

The procedure of EUS-GBD using LAMS depends on the presence of a cautery-enhanced tip. When using LAMS without the cautery-enhanced tip, the initial puncture of the gallbladder with a fine aspiration needle (usually 19 G) under the EUS guidance is performed. A guidewire (0.025 or 0.035 inch) is introduced through the needle under fluoroscopic control, making several loops inside the gallbladder. After removing the needle, a track dilatation is performed using a bugie dilator or balloon, usually up to a diameter of 4–5 mm. Finally, LAMS is introduced over the guidewire. A distal flange of LAMS is deployed under EUS guidance, followed by the proximal flange, usually under endoscopic control.

In recent years, however, predominantly used LAMS is the one with the cautery-enhanced tip. The advantage of this type is a direct introduction of the stent into the gallbladder without the need for a guidewire, track dilation, or fluoroscopy, which makes this method significantly convenient and much faster. This type of LAMS is fully covered and preloaded onto a catheter-based delivery system, with the cautery device at the tip [62]. 

In order to achieve optimal procedure control, the recommended distance between the puncture site and the gallbladder lumen is less than 10 mm. The inner diameter of LAMS used for gallbladder drainage is usually 10, 15, or 20 mm. The largest diameter allows for spontaneous or assisted evacuation of gallstones, as well as cholecystoscopy. 

Regarding the endoscopic approach (gastric or duodenal) to the gallbladder, the most important factor is to choose the spot with the closest distance from the gastrointestinal tract to the gallbladder wall. There is no significant difference in complication rates between gallbladder drainage from the stomach or duodenum [63]. However, there is a consideration that LAMS could be easily occluded by food particles, and stent migration could occur due to stronger gastric muscle contractions when the stent is positioned in the prepyloric antrum [64]. On the other hand, if subsequent cholecystectomy and the closure of the drainage fistula are indicated, the antrum is intraoperatively more accessible [65].

EUS-GBD performance requires extensive knowledge of hepatobiliary diseases and considerable experience in EUS. Since gallbladder drainage is technically more challenging than other EUS procedures, it is recommended that sufficient experience with LAMS drainage of peripancreatic collections is achieved (at least 10 procedures) before moving to EUS-GBD. [65]. As for getting experienced with EUS-GBD, according to literature reports, it requires 19 to 25 procedures to achieve sufficient competency [66,67].

Due to technical difficulties, moderate to deep sedation is required during the procedure [64].

#### 3.1.3. Duration

Regarding the postprocedural follow-up, a control endoscopy after 4–6 weeks is usually required. It is expected that by that time, more than half of patients will have spontaneous gallstone elimination through LAMS, while in the remaining patients, stone retrieval by endoscopic devices (baskets, lithotripsy) is applicable [57]. When the gallbladder is completely cleared from gallstones, LAMS can be removed without the need for long-term stenting. However, this approach carries the risk of recurrent cholelithiasis estimated at 40% and repeated development of AC [68]. 

The replacement of LAMS with the double pigtail plastic stent also presents further treatment options in order to create a long-term fistula. This replacement is needed since the long-term presence of LAMS may cause gallbladder mucosal abrasions and stent blockage with food particles [68,69]. The other option is to permanently leave LAMS in place. This approach is usually reserved for high-risk patients with substantial comorbidities, with a 3-year success rate of approximately 86% [54]. Regarding the postprocedural cholecystectomy, no difference has been found in the complication rates or conversion from laparoscopic to open cholecystectomy between the patients treated with the PGBD and EUS-GBD [70]. 

#### 3.1.4. Adverse Events and Outcomes 

Postprocedural complications are reported in 8–20% of patients who underwent EUS-GBD. The most common adverse effects are stent migration into the gallbladder or peritoneum, stent obstruction, bile leakage, bleeding, perforation, peritonitis, and pneumoperitoneum, as well as recurrent cholecystitis [68,71]. Stent displacement considers stent deployment in the stomach or duodenum or its migration into the peritoneum. Pneumoperitoneum is one of the most common complications, which may be reduced by using carbon dioxide for endoscopic insufflation [68]. Although some of these complications may be deleterious, the reported EUS-GBD-related mortality rate is 1–3.9% and is usually caused by sepsis [72,73]. Lisotti A et al. conducted a retrospective analysis that included 34 patients with AC treated with EUS-GBD. The reported 30-day and 1-year mortality were 12% and 32%. Severe comorbidities and acute kidney injury, and not the procedure success, were found as independent predictive factors of long-term mortality after EUS-GBD [74]. Fabbri et al. conducted a meta-analysis that included 1004 patients treated with EUS-GBD and concluded that physicians’ experience and anti-migrating devices were the main determinants of clinical outcomes [73].

### 3.2. Endoscopic Transpapillary Gallbladder Drainage

ET-GBD considers the placement of a nasobiliary drainage tube or plastic stent via the cystic duct into the gallbladder using the ERCP-guided transpapillary approach. It is a method that requires significantly developed endoscopic skills and should, therefore, be performed by experienced pancreatobiliary endoscopists in high-volume centers [7].

In 1984, Kozarek showed that cannulation of the cystic duct could be accomplished during ERCP, arguing that this may lead to better gallbladder visualization, retrieval of pure gallbladder bile for culture, as well as gallstone dissolution or extraction [75]. In the late 1980s, ERCP-guided gallbladder cannulation was used for direct application of the methyl-tert butyl ether (MTBE) that served for the dissolution of residual stone fragments after extracorporeal shock wave lithotripsy (ESWL). This course of treatment was later abandoned due to concerns about MTBE toxicity. However, a report of successful endoscopic transpapillary drainage in two patients with gallbladder empyema, which was published in 1990 by Feretis et al., was a cornerstone for the further development and implementation of ET-GBD [76]. 

#### 3.2.1. Indications and Contraindications

It is estimated that approximately 8–20% of patients with AC suffer from choledocholithiasis [77,78]. Therefore, if the ERCP is needed for the management of choledocholithiasis, ET-GBD may be chosen in extension as the drainage modality. Patients who have elevated biochemical cholestatic parameters should also be considered for ET-GBD since there is a great probability of choledocholithiasis even without clear imaging proof [79]. Contraindications include unstable cardiovascular status; structural abnormalities of the esophagus, stomach, or duodenum; as well as altered surgical anatomy, and coagulopathy (when the sphincterotomy is demanded). 

#### 3.2.2. Procedure

ET-GBD implies two different techniques. Both of them include the use of a duodenoscope, cannulation of papilla Vateri, and subsequent selective cannulation of the cystic duct. The following step ensures the placement of a guidewire through the cystic duct into the gallbladder, which is followed by the positioning of the elected drainage system over the guidewire.

The first method is endoscopic nasobiliary gallbladder drainage, which stands for the placement of a nasobiliary drainage tube (5–8.5 Fr) into the gallbladder. The advantage of this method is that one can see and follow the amount and quality of drainage content and also take repeated samples for microbiological analysis if needed. It is also possible to “flush” the drain and gallbladder itself through the nasogastric tube. The major benefit of this approach is that it does not necessarily require sphincterotomy and, thus, avoids all possible complications, such as bleeding or perforation. That being the case, this method is considered an adequate choice in the management of patients with AC who are taking antiaggregant or anticoagulant therapy or suffering from coagulopathy [7].

The other method is endoscopic gallbladder stenting, which results in the placement of biliary (usually 7–10 Fr double pigtail) stent from the gallbladder to the duodenum. In contrast to the aforementioned procedure, this approach must involve sphincterotomy. The advantage is that the patient does not suffer from the discomfort of the nasal tube placement. Furthermore, the patient cannot interrupt the drainage process by taking the drain out intentionally or unintentionally. Another advantage is that the stent can be kept in place for a longer period when compared to the nasobilliary drain, although there are still ongoing debates about the best timing for the stent or drain removal. One of the main concerns after successful stent placement is the possibility of stent occlusion and subsequent recurrent inflammation. Due to this issue, certain authors advocate the placement of two stents rather than only one [80]. 

Two randomized trials that compared these two methods did not show any significant difference in technical success rates, clinical outcomes, or adverse events [77,81]. 

#### 3.2.3. Duration

Long-term stenting seems to be the best option following transpapillary drainage since the stent keeps the cystic duct patent and, thereby, decompresses the gallbladder. In a study by Lee TH et al., 80% of patients were followed up for more than 12 months without developing any long-term complications, and the median stent patency was 760 days. Therefore, the authors concluded that ET-GBD may provide long-term stent patency without the need for scheduled stent exchanges [82].

#### 3.2.4. Adverse Events and Outcomes

Possible adverse events include stent migration, bleeding, injury, and the perforation of the duodenum, common bile duct or cystic duct, biliary leaks, cholangitis, and pancreatitis. The reported complication rates range from 5.4 to 19.3% [83,84,85]. The most commonly reported long-term adverse event is stent migration [85]. It is, thus, recommended to place the stent as distally as possible into the gallbladder since this will keep the secure position after the inflammation resolves [85]. Malik A et al. recently published a meta-analysis that included seven studies and a total of 335 patients with a reported complication rate of 5.4%. Postprocedural pancreatitis occurred in 3.5% of patients [83]. The important advantage of ET-GBT over EUS-GBD is the absence of postprocedural anatomical changes and consequent difficulties for surgical treatment. If the patient’s condition improves to such an extent that he becomes a candidate for surgical treatment, the stent may be easily removed [85]. The authors found no significant differences regarding the rates of postoperative complications or conversion to open cholecystectomy when compared with the PGBD [85,86]. 

## 4. Discussion

The modern development of medicine has contributed to a longer life expectancy and, thus, to an increasing number of high-aged patients suffering from a significant number of comorbidities. In this context, surgical treatment of AC stopped being a convenient method for a remarkable amount of patients and led clinicians to search for more conservative options. PGBD has, for a long time, been the treatment of choice for this group of patients and, according to TG18, is still recommended as the standard method. However, prompt development in the field of endoscopy that took place in the last decade contributed to new and attractive methods of gallbladder drainage, which, according to published studies, seem to be at least equally efficient as the PGBD. 

PGBD is a safe and effective alternative to the Lap-C that leads to clinical, laboratory, and radiological improvement. The annual costs for treatment of gallbladder disease are estimated at USD 6.5 billion in the United States, taking into mind that the early Lap-C has lower costs when compared with delayed Lap-C [87]. In 2018, Loozen CS et al. conducted the first randomized controlled trial that compared PGBD with the Lap-C. This large trial, named CHOCOLATE, was conducted in the Netherlands from 2011 to 2016 and included 142 high-risk patients with AC. The obtained results showed a high rate of major complications in the PGBD group (65% vs. 12%; risk ratio 0.19, *p* < 0.001), as well as a higher rate of recurrent biliary disease (53% vs. 5%, *p* < 0.001), and longer hospital stay (9 vs. 5 days, *p* < 0.001) [88]. In the spirit of these findings, TG18 stated that early Lap-C should be a choice of treatment rather than PGBD, followed by delayed surgery within one week from the symptom onset [89]. Recently, a meta-analysis that evaluated six studies and included a total of 8960 high-risk surgical patients found an increased mortality and readmission rate in patients treated with PGBD compared to the patients treated with emergency cholecystectomy [90]. However, in 2023, Cirocchi R et al. conducted a meta-analysis that included 32 studies with a total of 4188 patients (841 from RCTs and 3347 from no-RCTs) and found a lower incidence of postoperative complications in patients treated with PGBD before LC, thereby recommending preoperative PGBD in high-risk surgical patients or patients with longstanding AC [91]. 

The problem that seems to be escalating in recent years is clinicians bypassing cholecystectomy and preferring gallbladder drainage as a bridging or definitive treatment in borderline clinical indications. Studies show that the rate of patients who did not undergo surgical treatment after the PGBD ranges from 43% to 94% [92]. Ostapenko A et al. found a significant rise in the PGBD incidence from 2014 until 2018 (13.8% to 22.5%) [93]. 

When taking in mind all of the aforementioned findings, early cholecystectomy should be considered as the treatment of choice whenever possible. It offers a definitive treatment, lower complication rates, no disease recurrence, and is more cost-effective. However, if the GBD is indicated, the second controversy to discuss is which method to choose. 

According to the studies comparing PGBD with the EUS-GBD, the technical and clinical success rates of these two methods are comparable [58,59,60,61,94]. This finding, as well as improved patient outcomes in patients treated with the EUS-GBD, were confirmed in a recently published international randomized multicentre controlled trial (DRAC 1) that included 80 patients in the period from 2014 to 2018 [60]. Treatment with PGBD seems to be associated with higher postprocedural pain scores and longer hospitalization time [59,60,61]. Short-term and long-term complication rates, disease recurrence, and the need for reintervention are also reported in higher rates in the PGBD than in the EUS-GBD group [59,60,94]. Additionally, when compared to percutaneous techniques, endoscopic biliary drainage methods are also considered more cost-effective [95]. Nonetheless, EUS-GBD allows for the definitive treatment of cholecystolithiasis since the goal of this method is either the spontaneous elimination of gallstones or their retrieval using endoscopic devices. This fact usually implies bypassing cholecystectomy if the further disease course is not complicated by the gallstone recurrence. 

Iino C et al. retrospectively compared the efficacy and safety of ET-GBD and PGBD on 75 patients with AC and found PGBD to have significantly higher technical success rates (100% vs. 77%, *p* < 0.001) [96]. However, Itoi T et al. conducted a propensity score-matched analysis on 330 pairs of patients and found no significant differences in clinical success rates [97]. Both studies revealed no significant differences in the occurrence of complications but a significantly shorter median of drainage days and hospitalization duration in the ET-GBD group [96,97]. Studies also found significantly lower recurrence rates in the ET-GBD group when compared with the PGBD group (17% vs. 0%, *p* = 0.043) [98]. 

Regarding the effectiveness of endoscopic methods for gallbladder drainage, data from recent studies suggest that EUS-GBD has higher technical and clinical success rates than ET-GBD, although the overall mortality rate is lower in the ET-GBD group [72,99,100]. The rate of recurrent cholecystitis and a need for reintervention were found to be lower in the EUS-GBD group [99].

A recently published meta-analysis that included a total of 857 patients also found higher success rates in patients treated with EUS-GBD. The authors interpret the lower technical success rate of ET-GBD as a more challenging procedure due to obstacles (stones, strictures, metal stents) that obstruct the main biliary duct and the requisite of technically demanding cannulation of the cystic duct [101]. Additionally, the wide lumen of SEMS and LAMS allows for better biliary drainage and gallstone evacuation than plastic stents used via ERCP, thus enabling prompt clinical improvement and even the permanent resolution of the inflammation trigger that lies in gallstones [101]. They also found no statistically significant difference in complication rates between the methods [101]. Regarding the hemorrhagic adverse events, we already mentioned the benefits of ET-GBD with nasobiliary drainage. However, according to ESGE (European Society of Gastrointestinal Endoscopy), EUS-GBD appears to be a safe option in patients taking antithrombotics [102]. Although this statement needs to be confirmed in prospective trials before making the final recommendation, a study conducted by Ogura T et al. found no statistically significant difference in bleeding rates when comparing patients taking antiplatelets/anticoagulants with controls (7.3% vs. 2.6%, *p* = 0.33) [103].

Finally, Mohan BP et al. conducted a large meta-analysis that included a total of 15,131 patients and compared the efficacy of all three methods. They found the best clinical success rates in patients treated with the EUS-GBD (96.7% vs. 88.1% for ET-GBD vs. 89.3% for PGBD). Expectedly, ET-GBD had the lowest (83%) and PGBD had the highest (98.7%) technical success rates. Complication rates were similar among all of the methods [100].

It is still early to gather conclusions regarding the endoscopic drainage methods since they include new techniques implemented in a modest number of clinical centers. Additionally, there is a need for appropriate studies that would include patients who do not differ significantly from the population of interest, thereby avoiding selection biases.

## 5. Conclusions

Individualized approach and adjustment to the capabilities of the center where the patient is being treated are the two most important elements to consider when choosing among the gallbladder drainage methods in patients with AC. PGBD has the advantage of simplicity, as well as wide implementation in centers worldwide. Oppositely, endoscopic methods must rely on trained and high-skilled pancreatobiliary endoscopists with good technical support, but in reality, many centers lack these elements. EUS-GBD allows for definitive treatment due to adequate gallstone evacuation and, therefore, represents the method of choice in patients permanently unfit for surgery, while ET-GBD may serve as the bridging method. ET-GBD would be the first solution in patients with choledocholithiasis, while EUS-GBD and ET-GBD with nasobiliary drainage are appropriate options in patients taking antithrombotics.

## 6. Future Directions

Studies comparing the US-guided and MSCT-guided PGBD are lacking. EUS-GBD is currently considered a definitive treatment modality since the difficulty of cholecystectomy after EUS-GBD and the long-term outcomes of these patients are still not adequately evaluated. Additionally, there is still no clear proof that would define the necessity and frequency of stent replacements in patients treated with ET-GBD. Further randomized trials are needed to clarify these issues.

## Data Availability

Data are available upon request.
